# How Do Adults with Autism Spectrum Disorder Participate in the Labor Market? A German Multi-center Survey

**DOI:** 10.1007/s10803-021-05008-6

**Published:** 2021-04-17

**Authors:** Tolou Maslahati, Christian J. Bachmann, Juliana Höfer, Charlotte Küpper, Sanna Stroth, Nicole Wolff, Luise Poustka, Veit Roessner, Inge Kamp-Becker, Falk Hoffmann, Stefan Roepke

**Affiliations:** 1 Department of Psychiatry and Psychotherapy, Charité - Universitätsmedizin Berlin, Corporate Member of Freie Universität Berlin, Humboldt-Universität zu Berlin, Berlin Institute of Health, Campus Benjamin Franklin, Charité - Medical Faculty Berlin, Hindenburgdamm 30, 12203 Berlin, Germany; 2 Department of Child and Adolescent Psychiatry, Universitätsklinikum Ulm, Steinhövelstr. 5, 89075 Ulm, Germany; 3 Department of Health Services Research, Carl von Ossietzky University Oldenburg, Ammerländer Heerstraße 140, 26129 Oldenburg, Germany; 4 Department of Child and Adolescent Psychiatry, Philipps University Marburg, Hans-Sachs-Str. 4, 35039 Marburg, Germany; 5 Department of Child and Adolescent Psychiatry and Psychotherapy, University Medical Center Göttingen, Von-Siebold-Str. 5, 37075 Göttingen, Germany; 6 Department of Child and Adolescent Psychiatry, Medical Faculty of the Technical University Dresden, Fetscherstr. 74, 01307 Dresden, Germany; 7 Department of Psychology, Berlin School of Mind and Brain, Humboldt-Universität zu Berlin, Unter den Linden 6, 10099 Berlin, Germany

**Keywords:** Autism, Autism Spectrum Disorder, Labor Market, Education, Employment

## Abstract

International studies show disadvantages for adults with autism spectrum disorder (ASD) in the labor market. Data about their participation in the German labor market are scarce. The aim of this study was to examine the integration of adults with ASD in the German labor market in terms of education, employment and type of occupation by means of a cross-sectional-study, using a postal questionnaire. Findings show above average levels of education for adults with ASD compared to the general population of Germany and simultaneously, below average rates of employment and high rates of financial dependency. That indicates a poor integration of adults with ASD in the German labor market and emphasizes the need for vocational support policies for adults with ASD.

**Supplementary Information:**

The online version contains supplementary material available at 10.1007/s10803-021-05008-6.

Autism spectrum disorder (ASD) is a neurodevelopmental disorder with a chronic course and individuals with ASD face lifelong challenges (Lyall et al., 2017). The worldwide prevalence of ASD is just below 1% in the general population, with higher estimates in high-income countries (Lord et al., 2020). Adults with ASD show high rates of comorbid psychiatric disorders (Howlin & Magiati, 2017; Hudson et al., 2019), co-occurring intellectual disability (ID; Postorino et al., 2016) and impaired daily living skills (Bal et al., 2015). Further, they often experience poor social and functional outcomes (Sasson et al., 2020).

Even for adults with high-functioning autism (autism without ID), ASD can be a handicap in everyday work activities as the capacity to process socially relevant information and complex social interactions are often necessary requirements in occupational situations (Hedley et al., 2017; Martin et al., 2019). Obtaining and maintaining an employment is a challenge for adults with ASD and underemployment (jobs that underutilize their experience, knowledge and skills) is common (Chen et al., 2015; Hedley et al., 2017; Hillier & Galizzi, 2014; Roux et al., 2013). Possible barriers to an appropriate employment (appropriate to experience, knowledge and skills) of adults with ASD are difficulties with employers for example regarding the required support at work or the expected productivity level. In this context, difficulties of prioritization, self-organization, repetitive and maladaptive behavior (behavior that interferes with everyday activities) can hinder adults with ASD in the successful participation in the labor market (Howlin et al., 2004; Müller et al., 2003, 2008; Ohl et al., 2017). Studies from Australia (Baldwin et al., 2014) and from the USA (Hillier & Galizzi, 2014) showed, that adults with ASD face significant disadvantages in the labor market, despite their proficiency and willingness to work. Adults with ASD rather lose employment because of social interaction difficulties than because of their professional task performance (Hurlbutt & Chalmers, 2004).

Most studies on occupational activities of adults with ASD do neither differentiate between individuals with and without ID, nor do they consider severity of symptoms of ASD. Existing international studies show high rates of unemployment also in adults with ASD without ID (Baldwin et al., 2014). Howlin et al. (2013) found that 55% of adults with childhood autism without ID, have never had an employment and 72% of the individuals did not have formal educational qualifications. Other studies show higher educational levels and good prerequisites for a successful participation in the labor market, such as high school and university qualifications and an above average IQ (Hedley et al., 2017). Nevertheless, those individuals do not reach adequate employment status. Baldwin et al. (2014) showed that 46.2% of employed adults with ASD and no co-occurring ID were underemployed. Further studies showed that the employment rate of adults with ASD is significantly lower than rates of adults with other disabilities (including mental retardation and learning disabilities) and that salary is significantly lower for employed adults with ASD than for adults with other disabilities (Roux et al., 2013; Shattuck et al., 2012). Several studies indicate that most adults with ASD, who are enrolled in the labor market, only have part-time jobs, meaning they work less than 20 h a week (Baldwin et al., 2014; Migliore et al., 2012). Studies exploring sex differences in employment of adults with ASD did not find any overall differences between men and women on employment rates (Taylor et al., 2019).

International studies report rates up to 60% of unemployment for adults with ASD with and without intellectual disability (Hendricks, 2010; Howlin et al., 2013) and high risks of a disability pension for adults with ASD (Lallukka et al., 2020), but there is little knowledge about the participation of adults with ASD in the German labor market. The few hitherto existing studies on education and employment status of adults with ASD in Germany confirmed previous results, showing rates of unemployment of more than 40% (Lorenz & Heinitz, 2014; Proft, 2012) and disadvantages for adults with ASD regarding their participation in the labor market, especially in terms of unemployment, early retirement and underemployment (Frank et al., 2018; Riedel et al., 2016).

One of the largest societal cost components of supporting an individual with ASD in the UK and the USA is productivity loss as a result of lost or disrupted employment (Buescher et al., 2014). Furthermore, unemployment leads to a low quality of life in adults with ASD and is associated with social isolation, stress, mental health problems and an increased risk of homelessness (Baker et al., 2019; Kamio et al., 2013; Paul & Moser, 2009; Stone, 2019; Taylor & Hodapp, 2012).While the few mentioned studies are a first important examination of the employment status of adults with ASD in Germany, the samples consisted of clinically mostly late-diagnosed individuals, who predominately present high psychosocial adjustments only (Lehnhardt et al., 2012). Further, existing studies did not address adults with co-occurring ID in their surveys. While Riedel et al. (2016) and Lorenz and Heinitz (2014) only included adults with high-functioning autism, Frank et al. (2018) state their sample consists of “most likely not intellectually disabled individuals” only. Therefore, there is an urgent need for an adequate evaluation of employment status of adults with ASD with and without ID in Germany, in order to work out the importance for proper support for this population in the German labor market (Vogeley et al., 2013).

The aim of this study was to examine, by means of a cross-sectional-survey, the integration of a sample of clinically diagnosed adults with ASD with and without ID in the German labor market in terms of education, employment, type of occupation and potential sex differences. According to previous studies, we expect a poor integration into the labor market for adults with ASD in Germany.

## Methods

The present study is part of a large clinical and research network, the ASD-Net, focusing on the key challenges in ASD diagnostics, therapy and health service research, funded by the German Federal Ministry of Education and Research (Kamp-Becker et al., 2017).

### Recruitment and Participants

Between November 2015 and June 2016, four academic ASD outpatient clinics (including Clinics for Child and Adolescents Psychiatry) in Germany (Berlin, Dresden, Mannheim and Marburg) contacted all of their adult patients with a confirmed ASD diagnosis according to ICD-10 criteria (Dilling & Freyberger, 2012). All individuals were diagnosed by experienced clinicians using the current gold standard diagnostic procedures, the Autism Diagnostic Observation Schedule (ADOS) and—if parental informants were available—the Autism Diagnostic Interview—Revised (ADI-R). Individuals were included if they were 18 years or older and had a confirmed diagnosis of ASD according to ICD-10 (F84.0, F84.1, F84.5, F84.8, F84.9; (Dilling & Freyberger, 2012). The intellectual functioning of the individuals was measured in the different centers via the German version of the following assessments: Wechsler Intelligence Scale for Children (WISC-R; Tewes, 1983), WISC-III (Daseking et al., 2004), WISC-IV; (Franz Petermann & Petermann, 2011), Wechsler Adult Intelligence Scale (WAIS-R; Tewes, 1991), WAIS-III (Von Aster et al., 2006), Wechsler Preschool and Primary Scale of Intelligence (WPPSI-III; Petermann, Ricken, Fritz, Schuck, & Preuß, Petermann et al., 2014), Kaufman Assessment Battery for Children (Melchers & Preuß, 2009), Wortschatztest (Schmidt & Metzler, 1992), Raven’s Standard Progressive Matrices (Horn, 2009), and Raven’s Coloured Progressive Matrices (Bullheller, 2002). While ID is defined as an IQ < 70, some studies include borderline intellectual functioning (IQ = 71–84) (Baker & Blacher, 2020). According to ICD-10 (Dilling & Freyberger, 2012), an IQ = 71–85 is defined as a learning disability. Referring to that the level of intellectual functioning was classified into two groups in the current study: learning disability or intellectual impairment (IQ < 85) vs. no learning disability or intellectual impairment (IQ ≥ 85).

### Questionnaire and Data Collection

Based on the Client Service Receipt Inventory (CSRI; Knapp et al., 1999; Roick et al., 2001), one of the most commonly used measures of health and social service use, a questionnaire was developed and mailed to the participants. In exceptional cases, the questionnaire was handed over personally. The self-administered questionnaire was accompanied by a cover letter, a participant information sheet and a written informed consent form, in which participants were asked to consent on pseudonymized data linkage between their questionnaire data and some of their clinical data for instance age, sex, ICD 10-diagnosis, ADOS-2 comparison score and level of intellectual functioning. The written informed consent included the possibility for legal guardians and parents to fill in the questionnaire on behalf of the individuals with ASD. The level of intellectual functioning was divided into two groups: learning or intellectual disability (IQ < 85) vs. no learning or intellectual disability (IQ ≥ 85). We further summarized the ADOS-2 comparison score into the following three different groups: minimal to low (score 1–4), moderate (score 5–7), and high (score 9–10; Lord et al., 2012).

According to the CSRI, the questionnaire asked the participants about their employment status and their type of occupation in addition to demographic statistics. Two further questions generated data on the general and vocational education. The level of education was defined in accordance with the International Standard Classification of Education (ISCED, 2012; UNESCO, 1997) and classified into three groups: low (level 0–2B), medium (level 2A) and high education (level 3A). Referring to the German school system, low educational level complies with 9 years of schooling or leaving school without having acquired any school-leaving qualification. Medium educational level is equivalent to 10 years of schooling and high educational level complies with 12 or 13 years of schooling and a school-leaving qualification, which opens access to higher education institutions (Schneider, 2008; Schroedter et al., 2006). The ISCED was supplemented by a fourth category including participants still in education. The level of vocational education has been requested following the international Comparative Analysis of Social Mobility in Industrial Nations (CASMIN) classification (Brauns et al., 2003; Lechert et al., 2006) and was classified into three groups: low (no vocational qualification or other vocational qualification), medium (apprenticeship, vocational school) and high (University of applied science, University). Here as well, a fourth category included participants who were still in vocational education.

Data from the questionnaires were entered into an electronic Case Report Form (eCRF) created in OpenClinica® (OpenClinica Enterprise Version: 3.3) and were reviewed by a second person.

### Data Analysis

Baseline data were analyzed descriptively. Current employment status was evaluated stratified by diagnosis, vocational education and ID. Formal and vocational education level were analyzed descriptively with regard to diagnosis and intellectual functioning. The main source of income and type of financial support were also analyzed using descriptive statistics and were subdivided into each diagnostic category. Additionally, logistic regression was employed to detect associations between potential predictors and employment status. Statistical analyses were performed using SAS, version 9.4 (SAS Institute, Cary, NC, USA). Ethical approval for the study was obtained by the Commission for Research Impact Assessment and Ethics, Carl von Ossietzky University Oldenburg, and by the respective ethic committees of the participating study sites.

## Results

Survey documents were sent to 782 adults with ASD. Due to a wrong address, 52 mailings could not be delivered. 206 persons returned the questionnaires including a signed consent form (response: 26.8%). In 10 cases pseudonymized data linkage was not possible, leaving 196 questionnaires that could be evaluated.

The baseline characteristics are presented in Table 1 (Table 1). Of 196 respondents, 156 (79.6%) were male and the average age at the time of the survey was 31.5 years (standard deviation: 10.9; range: 18–67). Asperger syndrome (57.1%), childhood autism (27.6%) and atypical autism (11.7%) were the most frequent diagnoses in the sample. 68.9% of participants were of average or above-average intelligence.

**Table 1:** Baseline characteristics

	*n*	%
Sex (*n* = 196)		
Male	156	79.6
Female	40	20.4
Mean age, in years (± SD; range)	31.5 (10.9; 18–67)	
Age groups in years		
18–24	61	31.1
25–34	73	37.2
≥ 35	62	31.6
Diagnosis		
Childhood autism (F84.0)	54	27.6
Atypical autism (F84.1)	23	11.7
Asperger syndrome (F84.5)	112	57.1
Other specified pervasive developmental disorders (F84.8) and PDD-NOS (F84.9)	7	3.6
ADOS—2 comparison score		
Minimal till low (1–4)	36	22.4
Moderate (5–7)	65	40.4
High (8–10)	60	37.3
Intellectual functioning		
IQ ≥ 85	124	68.9
IQ < 85	56	31.1
Age at diagnosis in years		
< 18	96	49.7
> 18	97	50.3

### Level of Formal Education and Training

Regarding the general education level according to ISCED, 9.7% (*n* = 19) of all participants were classified into a low education level (German: *Hauptschulabschluss*, basic certificate of secondary education), 26.5% (*n* = 52) were classified into a medium education level (German: *Realschulabschluss*, secondary certificate of secondary education) and 43.9% (*n* = 86) were classified into a high level of education (German: *Fachhochschulreife* and *Hochschulreife*; general higher education entrance qualification). Of all participants 5.6% (*n* = 11) were still in education.

**Fig. 1:**
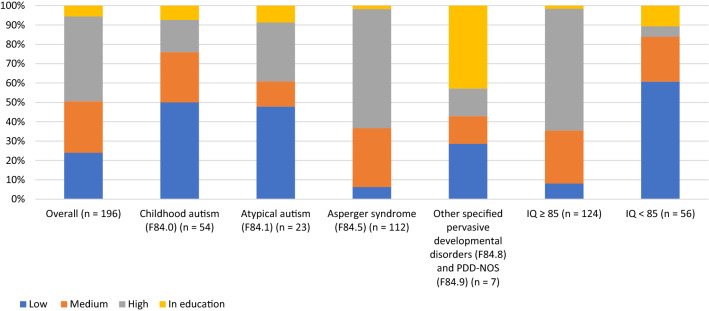
General education level of participants with regard to diagnosis and intellectual functioning. *Note.* The level of formal education was defined in accordance with the International Standard Classification of Education (ISCED) and was classified into three groups: low (ISCED level 0–2B), medium (level 2A) and high education (level 3A). A fourth category was supplemented including participants still in education

**Fig. 2:**
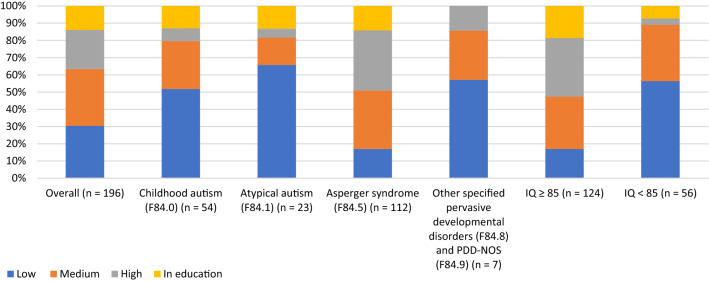
Vocational education level of participants with regard to diagnosis and intellectual functioning. *Note.* The level of vocational education was defined in accordance with the International Comparative Analysis of Social Mobility in Industrial Nations (CASMIN) classification and was classified into three groups: low (no vocational qualification or other vocational qualification), medium (German: *Lehre*, *Fachschule*; vocational school) and high (University diploma). A fourth group (In education) was supplemented

With regards to the vocational education and training according to CASMIN, 25.8% (*n* = 50) of the participants were assigned into a low level of vocational training (no vocational qualification or other vocational qualification), 33.0% (*n* = 74) were assigned into a medium level (German: *Lehre*, *Fachschule*; vocational school) and 22.7% (*n* = 44) were assigned to a high vocational education level (University diploma). 13.9% (*n* = 27) of the participants were still in vocational education. The general education level according to ISCED (Fig. 1) and the level of vocational education in accordance to CASMIN (Fig. 2) are presented in Figs. 1 and 2. The figures also depict the level of formal and vocational education subdivided into the four diagnoses childhood autism (F48.0), Atypical autism (F84.1), Asperger syndrome (F84.5), other pervasive developmental disorders (F84.8) and PDD-NOS (F84.9) and separated by intellectual functioning (IQ < 85 vs. IQ ≥ 85).

### Employment Status, Financial Support

Of all participants, 17.5% (*n* = 34) reported either being employed or self-employed at present with a mean of 32.8 weekly hours of work (*SD* = 8.19, missing data *n* = 12) and 21.1% (*n* = 41) reported working in a sheltered workshop. 15.0% (*n* = 29) were in early retirement for health reasons and 1.0% (*n* = 2) were in regular retirement. 23.7% (*n* = 46) of all participants were still in education and 17% (*n* = 33) stated they were unemployed at the moment. 81.8% (*n* = 27) of participants, who were unemployed at the moment, stated they have had an employment before. Of those participants who were unemployed or in early retirement for health reasons, 47.5% (n = 29) had a high level of formal education and 29.5% (*n* = 18) had a high level of vocational education. Table 2 represents the current employment status considering vocational education, diagnosis, intellectual functioning and gender (Table 2). Logistic regression revealed a tendency for a higher chance to be employed for female subjects. Nevertheless, this effect was statistically not significant (OR 1.25, 95% CI 0.6–2.6). Likewise, an IQ < 85 was not significantly predictive for employment status (OR 1.4, 95% CI 0.7–2.9).

**Table 2:** Current employment status in consideration of diagnosis, vocational education, intellectual functioning and gender

	Selfemployed or employed % (*n*)	Sheltered work % (*n*)	In education % (*n*)	Not employed % (*n*)	Retired for health reasons % (*n*)	Regular retirement/others % (*n*)
Total Sample						
Low (n = 85)	1.2 (1)	31.8 (27)	38.8 (33)	8.2 (7)	11.8 (10)	8.2 (7)
Medium (64)	21.9 (14)	20.3 (13)	10.9 (7)	18.8 (12)	6.3 (4)	21.9 (14)
High (45)	44.4 (20)	2.2 (1)	11.1 (5)	28.9 (13)	11.1 (5)	2.2 (1)
Childhood Autism (F84.0)						
Low (*n* = 34)	0.0 (0)	52.9 (18)	24.5 (8)	5.9 (2)	11.8 (4)	5.9 (2)
Medium (*n* = 15)	13.3 (2)	33.3 (5)	0.0 (0)	20.0 (3)	26.7 (4)	6.7 (1)
High (*n* = 4)	50.0 (2)	0.0 (0)	0.0 (0)	25.0 (1)	25.0 (1)	0.0 (0)
Atypical Autism (F84.1)						
Low (*n* = 14)	0.0 (0)	42.9 (6)	35.8 (5)	7.14 (1)	7.1 (1)	7.1 (1)
Medium (*n* = 6)	0.0 (0)	50.0 (3)	0.0 (0)	16.7 (1)	33.3 (2)	0.0 (0)
High (*n* = 1)	0.0 (0)	0.0 (0)	0.0 (0)	0.0 (0)	100.0 (1)	0.0 (0)
Asperger Syndrome (F84.5)						
Low (n = 33)	3.0 (1)	6.1 (2)	60.6 (20)	12.1 (4)	9.1 (3)	9.1 (3)
Medium (n = 41)	26.8 (11)	12.2 (5)	17.1 (7)	19.5 (8)	17.1 (7)	7.3 (3)
High (n = 37)	43.2 (16)	2.7 (1)	13.5 (5)	29.7 (11)	8.1 (3)	2.7 (1)
Other Specified Pervasive Developmental Disorders (F84.8) and PDD-NOS (F84.9)						
Low (*n* = 4)	0.0 (0)	25.0 (1)	0.0 (0)	25.0 (1)	50.0 (2)	0.0 (0)
Medium (n = 2)	0.0 (0)	0 (0)	0.0 (0)	50.0 (1)	50.0 (1)	0.0 (0)
High (n = 1)	100.0 (1)	0 (0)	0.0 (0)	0.0 (0)	0.0 (0)	0.0 (0)
Intellectual Functioning						
IQ ≥ 85 (n = 124)	23.6 (29)	9.8 (12)	30.9 (38)	17.9 (22)	14.6 (18)	3.2 (4)
IQ < 85 (n = 56)	9.1 (5)	47.3 (26)	12.7 (7)	12.7 (7)	10.9 (6)	7.3 (4)
Gender						
Female (n = 40)	27.5 (11)	20.0 (8)	20.0 (8)	10 (4)	17.5 (7)	5.0 (2)
Male (n = 154)	14.9 (23)	21.4 (33)	24.7 (38)	18.8 (29)	14.3 (22)	5.8 (9)

Level of vocational education classified into three groups according to the International Comparative Analysis of Social Mobility in Industrial Nations (CASMIN): low (no vocational qualification or other vocational qualification), medium (German: *Lehre*, *Fachschule*; vocational school) and high (University diploma)

23.7% of all participants stated, their primary source of income is from salary. 63.4% of all participants depended on either public or familiar financial support. The main source of income subdivided into diagnosis is represented in Table 1 in the supplement. 72.8% of all participants stated that they receive some kind of public financial support (c.f. Table 2 in the supplement).

### Type of Occupation

Regarding the type of occupation, 6.1% (*n* = 5) of all working participants were self-employed, 26.8% (*n* = 22) of the participants worked as simple employees, 4.9% (*n* = 4) worked as managing employees, 6.1% (*n* = 5) worked as unskilled laborers, 12.2% (*n* = 10) worked as semi-skilled laborers and 6.1% (*n* = 5) were employed as skilled workers.

## Discussion

This study aimed to examine the participation of clinically diagnosed adults with ASD with and without intellectual impairment in the German labor market via a postal cross-sectional survey in former patients of four academic ASD outpatient clinics in Germany.

Regarding formal education level, the percentage of general higher education entrance qualification was higher in our study sample (43.9%) compared to the general population of Germany (30.1%, assessed in 2016; RDC of the Federal Statistical Office and Statistical Offices of the Federal States, 2017). Further, 22.7% of the participants possessed an university degree as their highest vocational qualification, which is higher compared to the general population in Germany in 2016, with 17.0% possessing an university degree (RDC of the Federal Statistical Office and Statistical Offices of the Federal States, 2017). However, compared to previous studies on ASD in German adults, the percentage of general higher education entrance qualification and university degree is rather low in the current sample (Frank et al., 2018; Riedel et al., 2016). Existing studies in Germany report rates of at least 52% for general higher education entrance qualification and rates of 37.6% and higher for university diplomas in adults with ASD (Frank et al., 2018; Riedel et al., 2016). This can be explained by differences of sample characteristics in the selected ASD populations. Previous studies included mostly late-diagnosed adults with ASD and no co-occurring ID only (Frank et al., 2018; Lorenz & Heinitz, 2014; Riedel et al., 2016). Although the current sample was also not representative for individuals within the whole spectrum of ASD (as discussed below), it draws on a more diverse sample by including adults with ID.

Education level has been shown to be predictive for employment status in adults with ASD (Ohl et al., 2017). According to this, chances to participate in the German labor market seem to be promising for the present sample. Nevertheless, only 32.1% of the participants state to have been employed at some point in their life and only 17.5% of the sample state to be enrolled in the regular job market at the time of the survey. The unemployment rate was 17.0%, which is alarmingly high considering the rate for the general population in Germany in 2016 was 6.1% only (RDC of the Federal Statistical Office and Statistical Offices of the Federal States, 2017). The poor participation in the labor market of our sample does not only underreach the general population of Germany, but also hitherto existing data on the participation of adults with ASD in Germany (Frank et al., 2018; Riedel et al., 2016). This again can be explained by the different composition of analyzed samples as our study included a broader spectrum of individuals with ASD than existing studies in Germany. Compared to international studies, reporting unemployment rates of 50% and higher for adults with ASD (Hendricks, 2010), the rate of 17% in the current study seems oddly low. This may partly be due to methodological reasons. While international studies have assessed employment status dichotomously (employed vs. unemployed), the current study provides further subdivision. Adding up the subcategories (unemployed, early retirement and retirement), this study reveals an unemployment rate of 37.6%. The sustaining lower unemployment rate compared to international studies may be a result of adults with more severe symptoms being underrepresented in this current study. Another indicator of the poor integration in the labor market is the high percentage of financial dependency of the individuals in this sample. 72.8% of the participants receive some kind of financial support and more than two thirds even depend on either public or familiar financial support.

As in former international studies (Baldwin et al., 2014; Hillier & Galizzi, 2014) and in previous studies in Germany (Frank et al., 2018; Riedel et al., 2016), our findings show above average levels of formal and vocational education in adults with ASD and at the same time below average integration in the labor market. Existing studies investigating the effect of other psychiatric disorders on labor market outcomes also show an adverse effect on employment status (Banerjee et al., 2017; Hakulinen et al., 2019a). What is different in adults with ASD is the high level of education. Among impairments in the labor market, other psychiatric disorders, such as schizophrenia or mood disorders, are also associated with lower levels of education (Hakulinen et al., 2019b, c). The present ASD sample, however, shows above average levels of education. Nevertheless, the rates of employment are well below average compared to the general population. The unemployed ratio for participants with ASD and a high level of education was 20.3%. For comparison, the ratio in the general German population for adults with a high level of education was 2.0% (RDC of the Federal Statistical Office and Statistical Offices of the Federal States, 2017). Apparently, adults with ASD face challenges obtaining an employment, despite high levels of education. This is alarming and requires explanation. Comorbid psychiatric diagnoses, which have been shown to be common in adults with ASD (Howlin & Magiati, 2017; Hudson et al., 2019; Noterdaeme & Wriedt, 2010; Strunz et al., 2014), may explain the poor integration in the labor market to a certain degree. However, as discussed below, comorbid psychiatric diagnoses were not included in the analyses of this current paper. Their impact can therefore not be evaluated. It can further be presumed that impairments associated with ASD, such as impaired executive and soft skills, may rather be tolerated in educational environments than in the labor market. Moreover, educational environments may provide more assistance than potential employers may provide. Besides that, working environments often require more social skills, which are impaired in adults with ASD (Müller et al., 2008). The combination of less assistance and more requirements in the labor market may explain the higher attainment of individuals with ASD in educational institutions than in the labor market. This emphasizes the need to support adults with ASD to fulfill their potential and to better integrate them in the German labor market. First international programs for on-the-job coaching activations indicate to be effective for adults with ASD (Mawhood & Howlin, 1999; Vogeley et al., 2013; Wehman et al., 2014), but it remains unclear if those programs are needed for all individuals with ASD. Transition programs, that aim at preparing young adults with ASD to find and achieve employment by focusing on social communication and independent living skills, have also been shown to be effective (Cimera et al., 2013) and have been recommended (Solomon, 2020). The need for the implementation of such programs in Germany should be further assessed in future investigations. However, recent findings also emphasize the need for environmental changes in order to improve employment outcomes for adults with ASD (Black et al., 2019). For one, potential strengths associated with ASD should be recognized and enhanced in occupational settings (Wong et al., 2018). This could be accomplished by implementing ASD specific employer and/or co-worker trainings. Black et al. (2019) showed that autism education and awareness trainings enabled employers and co-workers to recognize ASD associated strengths and significantly facilitated work processes for adults with ASD. Further implementing support systems in the working environment, by for example appointing a designated supervisor or peer mentor, has been identified as an important strategy to support employees with ASD to manage their workplace concerns and maximize their potential (Dreaver et al., 2020; Nicholas et al., 2018). Finally, hiring processes should be accommodated in order to detect strengths and work related competencies instead of only evaluating social and communication skills (Austin & Pisano, 2017; Griffiths et al., 2020).

Research should specifically address difficulties and general experiences of adults with ASD in professional transitions and in the labor market.

## Strength and Limitations

A main strength of this current study is the accurateness of diagnosis of the participants, as all of them have been diagnosed by specialized outpatient clinics based at university hospitals (Kamp-Becker et al., 2018). At the same time, recruitment through specialized ASD clinics may be a limitation of the study, because the sample may be biased towards a help seeking population. It has further been shown that access to diagnostic services is associated with higher education (Smith et al., 2020). The sample composition may therefore not be representative for the general population of adults with ASD in Germany. Representativeness may further be a critical issue of this study, because the response rate was not high (26.8%). It is reasonable to assume that individuals with more severe symptoms did not participate in this survey. That may be represented by the prevalence of learning or intellectual disability of only 31.1% in this sample, which is lower than in other studies, reporting prevalence rates of at least 40.0% and higher (Charman et al., 2011; Noterdaeme & Wriedt, 2010; Postorino et al., 2016). It is most probable that individuals with intellectual impairment were not able to fill in the questionnaire and therefore could not participate in the survey. Hence, they are underrepresented in the current study.

The proportion of male and female participants in our study is consistent with the previously reported male/female ratio in ASD (Fombonne et al., 2009). However, it has become a matter of debate whether females are underdiagnosed and whether diagnostic instruments are sufficient to detect the postulated female autism phenotype that seems to differ from a male autism phenotype (Lai & Szatmari, 2020).

As participants from different outpatient clinics were included in this survey, the diagnostic of comorbid psychiatric disorders varied, depending on the respective recruitment center and was not standardized. Therefore, comorbid psychiatric diagnoses were not entirely comparable and were not included in the analyses of the current study. Given that comorbid psychiatric disorders are common in adults with ASD (Howlin & Magiati, 2017; Hudson et al., 2019; Noterdaeme & Wriedt, 2010; Strunz et al., 2014), this may have had an impact on the employment status of the sample and cannot be evaluated with the available data. Further, maladaptive and repetitive behavior, which have been shown to influence the employment status of adults with ASD (Howlin et al., 2004), were not assessed in this study. That may have influenced the results and has to be taken into consideration.

## Conclusion

This study shows high levels of formal and vocational education for adults with ASD, exceeding the level of the general population in Germany. At the same time, their participation in the labor market underreach the participation of the general population. Despite their high education level, two thirds of the adults with ASD in this sample depend on either public or familiar financial support and show high rates of unemployment. Accordingly, adults with ASD are poorly integrated in the German labor market. To enhance the participation of adults with ASD, on-the-job coaching, transition programs and environmental adjustments in the labor market have shown promising effects in prior studies. Results of this study and findings of previous studies emphasize the further need to implicate those programs and environmental changes in Germany.

## Supplementary Information

Below is the link to the electronic supplementary material.

Supplementary file1 (DOCX 22 KB)

## References

[CR2] Austin RD, Pisano GP (2017). Neurodiversity as a competitive advantage. Harvard Business Review.

[CR3] Baker BL, Blacher J (2020). Brief report: Behavior disorders and social skills in adolescents with autism spectrum disorder: Does IQ matter?. Journal of Autism and Developmental Disorders.

[CR4] Baker EK, Richdale AL, Hazi A (2019). Employment status is related to sleep problems in adults with autism spectrum disorder and no comorbid intellectual impairment. Autism.

[CR5] Bal VH, Kim S-H, Cheong D, Lord C (2015). Daily living skills in individuals with autism spectrum disorder from 2 to 21 years of age. Autism.

[CR6] Baldwin S, Costley D, Warren A (2014). Employment activities and experiences of adults with high-functioning autism and Asperger’s disorder. Journal of Autism and Developmental Disorders.

[CR100] Banerjee, S., Chatterji, P., & Lahiri, K. (2017). Effects of psychiatric disorders on labor market outcomes: a latent variable approach using multiple clinical indicators. *Health Economics, 26*(2), 184–205. 10.1002/hec.3286 26563992

[CR7] Black MH, Mahdi S, Milbourn B, Thompson C, D'Angelo A, Ström E, Falkmer M, Falkmer T, Lerner M, Gerber A, Halladay A (2019). Perspectives of key stakeholders on employment of autistic adults across the United States, Australia, and Sweden. Autism Research.

[CR8] Brauns, H., Scherer, S., & Steinmann, S. (2003). The CASMIN educational classification in international comparative research. In *Advances in cross-national comparison* (pp. 221–244). Springer.

[CR9] Buescher AV, Cidav Z, Knapp M, Mandell DS (2014). Costs of autism spectrum disorders in the United Kingdom and the United States. JAMA Pediatrics.

[CR10] Bullheller, S. (2002). *Matrices HHOCP.(CPM).(Deutsche Bearbeitung und Normierung nach JC Raven)*. Pearson Assessment.

[CR11] Charman T, Pickles A, Simonoff E, Chandler S, Loucas T, Baird G (2011). IQ in children with autism spectrum disorders: Data from the Special Needs and Autism Project (SNAP). Psychological Medicine.

[CR12] Chen JL, Leader G, Sung C, Leahy M (2015). Trends in employment for individuals with autism spectrum disorder: A review of the research literature. Review Journal of Autism and Developmental Disorders.

[CR13] Cimera RE, Burgess S, Wiley A (2013). Does providing transition services early enable students with ASD to achieve better vocational outcomes as adults?. Research and Practice for Persons with Severe Disabilities.

[CR14] Daseking M, Petermann F, Tewes U, Rossmann P, Schallberger U (2004). Hamburg-Wechsler-Intelligenztest für Kinder III (HAWIK-III). Kindheit und Entwicklung.

[CR15] Dilling H, Freyberger HJ (2012). Taschenführer zur ICD-10-Klassifikation psychischer Störungen.

[CR16] Dreaver J, Thompson C, Girdler S, Adolfsson M, Black MH, Falkmer M (2020). Success factors enabling employment for adults on the autism spectrum from employers’ perspective. Journal of Autism and Developmental Disorders.

[CR17] Fombonne E, Quirke S, Hagen A (2009). Prevalence and interpretation of recent trends in rates of pervasive developmental disorders. McGill Journal of Medicine: MJM.

[CR18] Frank F, Jablotschkin M, Arthen T, Riedel A, Fangmeier T, Hölzel LP, van Elst LT (2018). Education and employment status of adults with autism spectrum disorders in Germany—A cross-sectional-survey. BMC Psychiatry.

[CR19] Griffiths AJ, Hanson AH, Giannantonio CM, Mathur SK, Hyde K, Linstead E (2020). Developing employment environments where individuals with ASD Thrive: Using machine learning to explore employer policies and practices. Brain Sciences.

[CR101] Hakulinen, C., Elovainio, M., Arffman, M., Lumme, S., Pirkola, S., Keskimäki, I., Manderbacka, K. & Böckerman, P. (2019a). Mental disorders and longterm labour market outcomes: nationwide cohort study of 2 055 720 individuals. *Acta Psychiatrica Scandinavica, 140*(4), 371–381. 10.1111/acps.13067 31254386

[CR102] Hakulinen, C., McGrath, J. J., Timmerman, A., Skipper, N., Mortensen, P. B., Pedersen, C. B., & Agerbo, E. (2019b). The association between early-onset schizophrenia with employment, income, education, and cohabitation status: nationwide study with 35 years of follow-up. *Social Psychiatry and Psychiatric Epidemiology, 54*(11), 1343–1351. 10.1007/s00127-019-01756-0 31456027

[CR103] Hakulinen, C., Musliner, K. L., & Agerbo, E. (2019). Bipolar disorder and depression in early adulthood and long‐term employment, income, and educational attainment: A nationwide cohort study of 2,390,127 individuals. *Depression and Anxiety, 36*(11), 1080–1088. 10.1002/da.22956 31508865

[CR20] Hedley D, Uljarević M, Cameron L, Halder S, Richdale A, Dissanayake C (2017). Employment programmes and interventions targeting adults with autism spectrum disorder: A systematic review of the literature. Autism.

[CR21] Hendricks D (2010). Employment and adults with autism spectrum disorders: Challenges and strategies for success. Journal of Vocational Rehabilitation.

[CR22] Hillier, A., & Galizzi, M. (2014). Employment outcomes for young adults with autism spectrum disorders. *Review of Disability Studies: An International Journal, 10*(1&2). http://hdl.handle.net/10125/58586

[CR23] Horn R (2009). Standard Progressive Matrices (SPM). Deutsche Bearbeitung und Normierung nach JC Raven.

[CR24] Howlin P, Goode S, Hutton J, Rutter M (2004). Adult outcome for children with autism. Journal of Child Psychology and Psychiatry.

[CR25] Howlin P, Magiati I (2017). Autism spectrum disorder: Outcomes in adulthood. Current Opinion in Psychiatry.

[CR26] Howlin, P., Moss, P., Savage, S., & Rutter, M. (2013). Social outcomes in mid-to later adulthood among individuals diagnosed with autism and average nonverbal IQ as children. *Journal of the American Academy of Child & Adolescent Psychiatry, 52*(6), 572–581.e571. 10.1016/j.jaac.2013.02.017 23702446

[CR27] Hudson CC, Hall L, Harkness KL (2019). Prevalence of depressive disorders in individuals with autism spectrum disorder: A meta-analysis. Journal of Abnormal Child Psychology.

[CR28] Hurlbutt K, Chalmers L (2004). Employment and adults with Asperger syndrome. Focus on Autism and Other Developmental Disabilities.

[CR29] ISCED, U. (2012). International standard classification of education 2011. *UNESCO Institute for Statistics*.

[CR30] Kamio Y, Inada N, Koyama T (2013). A nationwide survey on quality of life and associated factors of adults with high-functioning autism spectrum disorders. Autism.

[CR31] Kamp-Becker I, Albertowski K, Becker J, Ghahreman M, Langmann A, Mingebach T, Poustka L, Weber L, Schmidt H, Stehr T, Smidt J (2018). Diagnostic accuracy of the ADOS and ADOS-2 in clinical practice. European Child & Adolescent Psychiatry.

[CR32] Kamp-Becker I, Poustka L, Bachmann C, Ehrlich S, Hoffmann F, Kanske P, Kirsch P, Krach S, Paulus FM, Rietschel M, Roepke S, Roessner V, Schad-Hansjosten T, Singer T, Stroth S, Witt S, Wermter AK (2017). Study protocol of the ASD-Net, the German research consortium for the study of autism spectrum disorder across the lifespan: From a better etiological understanding, through valid diagnosis, to more effective health care. BMC Psychiatry.

[CR33] Knapp M, Scott S, Davies J (1999). The cost of antisocial behaviour in younger children. Clinical Child Psychology and Psychiatry.

[CR35] Lai MC, Szatmari P (2020). Sex and gender impacts on the behavioural presentation and recognition of autism. Current Opinion in Psychiatry.

[CR36] Lallukka T, Mittendorfer-Rutz E, Ervasti J, Alexanderson K, Virtanen M (2020). Unemployment trajectories and the early risk of disability pension among young people with and without autism spectrum disorder: A Nationwide Study in Sweden. International Journal of Environmental Research and Public Health.

[CR37] Lechert, Y., Schroedter, J. H., & Lüttinger, P. (2006). Die Umsetzung der Bildungsklassifikation CASMIN für die Volkszählung 1970, die Mikrozensus-Zusatzerhebung 1971 und die Mikrozensen 1976–2004.

[CR38] Lehnhardt, F.-G., Gawronski, A., Volpert, K. u., Schilbach, L., Tepest, R., & Vogeley, K. (2012). Das psychosoziale funktionsniveau spätdiagnostizierter patienten mit autismus-spektrum-störungen–eine retrospektive untersuchung im erwachsenenalter. *Fortschritte der Neurologie·Psychiatrie, 80*(02), 88–97. 10.1055/s-0031-1281642 22086712

[CR39] Lord C, Brugha TS, Charman T, Cusack J, Dumas G, Frazier T, Jones EJ, Jones RM, Pickles A, Lounds TJ, Veenstra-VanderWeele J (2020). Autism spectrum disorder. Nat Rev Dis Primers.

[CR40] Lord, C., Rutter, M., DiLavore, P., Risi, S., Gotham, K., & Bishop, S. (2012). Autism diagnostic observation schedule 2nd edition manual. *Western Psychological Services*.

[CR41] Lorenz T, Heinitz K (2014). Aspergers–different, not less: Occupational strengths and job interests of individuals with Asperger's syndrome. PLoS ONE.

[CR42] Lyall K, Croen L, Daniels J, Fallin MD, Ladd-Acosta C, Lee BK, Park BY, Snyder NW, Schendel D, Volk H (2017). The changing epidemiology of autism spectrum disorders. Annual Review of Public Health.

[CR43] Martin N, Barnham C, Krupa J (2019). Identifying and addressing barriers to employment of autistic adults. Journal of Inclusive Practice in Further and Higher Education.

[CR44] Mawhood L, Howlin P (1999). The outcome of a supported employment scheme for high-functioning adults with autism or Asperger syndrome. Autism.

[CR45] Melchers, P., & Preuß, U. (2009). Kaufman assessment battery for children (deutsche Version). *Pearson Assessment*.

[CR46] Migliore A, Timmons J, Butterworth J, Lugas J (2012). Predictors of employment and postsecondary education of youth with autism. Rehabilitation Counseling Bulletin.

[CR47] Müller E, Schuler A, Burton BA, Yates GB (2003). Meeting the vocational support needs of individuals with Asperger syndrome and other autism spectrum disabilities. Journal of Vocational Rehabilitation.

[CR48] Müller E, Schuler A, Yates GB (2008). Social challenges and supports from the perspective of individuals with Asperger syndrome and other autism spectrum disabilities. Autism.

[CR49] Nicholas DB, Mitchell W, Dudley C, Clarke M, Zulla R (2018). An ecosystem approach to employment and autism spectrum disorder. Journal of Autism and Developmental Disorders.

[CR50] Noterdaeme MA, Wriedt E (2010). Begleitsymptomatik bei tief greifenden Entwicklungsstörungen. Zeitschrift für Kinder-und Jugendpsychiatrie und Psychotherapie.

[CR51] Ohl A, Grice Sheff M, Small S, Nguyen J, Paskor K, Zanjirian A (2017). Predictors of employment status among adults with autism spectrum disorder. Work.

[CR52] Paul KI, Moser K (2009). Unemployment impairs mental health: Meta-analyses. Journal of Vocational Behavior.

[CR53] Petermann, F., & Petermann, U. (2011). Wechsler Intelligence Scale for Children®–Fourth Edition. *Pearson Assessment*.

[CR54] Petermann, F., Ricken, G., Fritz, A., Schuck, K., & Preuß, U. (2014). Wechsler Preschool and Primary Scale; Deutschsprachige Adaption nach D. Wechsler (3. überarbeitete und erweiterte Aufl.). *Pearson Assessment*

[CR55] Postorino V, Fatta LM, Sanges V, Giovagnoli G, De Peppo L, Vicari S, Mazzone L (2016). Intellectual disability in autism spectrum disorder: Investigation of prevalence in an Italian sample of children and adolescents. Research in Developmental Disabilities.

[CR56] Proft, J. (2012). Die berufliche Teilhabe von hochfunktionalen erwachsenen Personen mit einer Autismus-Spektrum-Störung auf dem ersten Arbeitsmarkt und ihre Erwartungen an einen Arbeitsplatz. *Bachelor Thesis, University of Cologne*.

[CR57] RDC of the Federal Statistical Office and Statistical Offices of the Federal States, [Internationale Bildungsindikatoren im Ländervergleich] [2017]. 10.3278/6001820fw

[CR58] Riedel A, Schröck C, Ebert D, Fangmeier T, Bubl E, van Elst LT (2016). Überdurchschnittlich ausgebildete Arbeitslose-Bildung, Beschäftigungsverhältnisse und Komorbiditäten bei Erwachsenen mit hochfunktionalem Autismus in Deutschland. Psychiatrische Praxis.

[CR59] Roick, C., Kilian, R., Matschinger, H., Bernert, S., Mory, C., & Angermeyer, M. C. (2001). Die deutsche Version des Client Sociodemographic and Service Receipt Inventory. *Psychiatrische Praxis, 28*(Sup. 2), 84–90. 10.1055/s-2001-17790 11605129

[CR60] Roux AM, Shattuck PT, Cooper BP, Anderson KA, Wagner M, Narendorf SC (2013). Postsecondary employment experiences among young adults with an autism spectrum disorder. Journal of the American Academy of Child & Adolescent Psychiatry.

[CR61] Sasson NJ, Morrison KE, Kelsven S, Pinkham AE (2020). Social cognition as a predictor of functional and social skills in autistic adults without intellectual disability. Autism Research.

[CR62] Schmidt, K., & Metzler, P. (1992). Wortschatztest (WST). Beltz Test GmbH.

[CR63] Schneider, S. L. (2008). *The international standard classification of education (ISCED-97): An evaluation of content and criterion validity for 15 European countries*. MZES.

[CR64] Schroedter, J. H., Lechert, Y., & Lüttinger, P. (2006). Die Umsetzung der Bildungsskala ISCED-1997 für die Volkszählung 1970, die Mikrozensus-Zusatzerhebung 1971 und die Mikrozensen 1976–2004 (Version 1).

[CR65] Shattuck PT, Narendorf SC, Cooper B, Sterzing PR, Wagner M, Taylor JL (2012). Postsecondary education and employment among youth with an autism spectrum disorder. Pediatrics.

[CR66] Smith KA, Gehricke J-G, Iadarola S, Wolfe A, Kuhlthau KA (2020). Disparities in service use among children with autism: A systematic review. Pediatrics.

[CR67] Solomon C (2020). Autism and employment: Implications for employers and adults with ASD. Journal of Autism and Developmental Disorders.

[CR68] Stone B (2019). ‘The domino effect’: Pathways in and out of homelessness for autistic adults. Disability & Society.

[CR69] Strunz, S., Dziobek, I., & Roepke, S. (2014). Komorbide psychiatrische Störungen und Differenzialdiagnostik bei nicht-intelligenzgeminderten Erwachsenen mit Autismus-Spektrum-Störung. *PPmP-Psychotherapie·Psychosomatik·Medizinische Psychologie, 64*(06), 206–213. 10.1055/s-0033-1358708 24234289

[CR70] Taylor JL, Hodapp RM (2012). Doing nothing: Adults with disabilities with no daily activities and their siblings. American Journal on Intellectual and Developmental Disabilities.

[CR71] Taylor JL, Smith DaWalt L, Marvin AR, Law JK, Lipkin P (2019). Sex differences in employment and supports for adults with autism spectrum disorder. Autism.

[CR72] Tewes U (1983). HAWIK-R: Hamburg-Wechsler Intelligenztest für Kinder-Revision: 1983.

[CR73] Tewes, U. (1991). *Hamburg-Wechsler Intelligenztest für Erwachsene: HAWIE-R*. Huber.

[CR74] UNESCO, M. (1997). *International standard classification of education: ISCED 1997 (re-edition)*. UNESCO-UIS. http://www.uis.unesco.org/TEMPLATE/pdf/isced

[CR75] Vogeley K, Kirchner J, Gawronski A, van Elst LT, Dziobek I (2013). Toward the development of a supported employment program for individuals with high-functioning autism in Germany. European Archives of Psychiatry and Clinical Neuroscience.

[CR76] Von Aster M, Neubauer A, Horn R (2006). Hamburg-Wechsler-Intelligenz-Test für Erwachsene III.

[CR77] Wehman PH, Schall CM, McDonough J, Kregel J, Brooke V, Molinelli A, Ham W, Graham CW, Riehle JE, Collins HT (2014). Competitive employment for youth with autism spectrum disorders: Early results from a randomized clinical trial. Journal of Autism and Developmental Disorders.

[CR78] Wong PS, Donelly M, Neck PA, Boyd B (2018). Positive autism: Investigation of workplace characteristics leading to a strengths-based approach to employment of people with autism. Revista de Management Comparat International.

