# Supplementary material for: How Do Adults with Autism Spectrum Disorder Participate in the Labor Market? A German Multi-center Survey
Source: J Autism Dev Disord. 2021 Apr 17;52(3):1066–76. doi: 10.1007/s10803-021-05008-6 (PMC8854283; doi:10.1007/s10803-021-05008-6)
Supplement: Supplementary file 1 — Supplementary file1 (DOCX 22 KB) [file 10803_2021_5008_MOESM1_ESM.docx]

Supplementary Material

How do Adults with Autism Spectrum Disorder Participate in the Labor Market? A German Multi-Center Survey

Journal of Autism and Developmental Disorder

Corresponding author:

Tolou Maslahati K.

Department of Psychiatry and Psychotherapy,

Charité - Universitätsmedizin Berlin, Corporate Member of Freie Universität Berlin, Humboldt-Universität zu Berlin, Berlin Institute of Health

E-mail: [Tolou.maslahati@charite.de](mailto:Tolou.maslahati@charite.de)

Phone: 0049 30 450 517567

**Table 1**

Main Source of Income with Regards to Diagnosis

|  | Childhood Autism (F84.0)  % (n) | Atypical Autism (F84.1)  % (n) | Asperger Syndrome (F84.5)  % (n) | Other Pervasive Developmental Disorders (F84.8); PDD-NOS (F84.9)  % (n) |
| --- | --- | --- | --- | --- |
| Income from work | 2.7 (5) | 1.1 (2) | 19.4 (36) | 0.5 (1) |
| Public financial support | 16.1(30) | 4.3 (8) | 14.5 (27) | 2.2 (4) |
| Familiar financial support | 7.0 (13) | 3.8 (7) | 15.6 (29) | 0.0 (0) |
| Pension or other | 2.2 (4) | 2.2 (4) | 7.5 (14) | 1.1 (2) |

**Table 2**

Type of Financial Support Subdivided into Four Diagnoses

|  | Childhood Autism (F84.0)  % (n) | Atypical Autism (F84.1)  % (n) | Asperger Syndrome (F84.5)  % (n) | Other Pervasive Developmental Disorders (F84.8); PDD-NOS (F84.9)  % (n) |
| --- | --- | --- | --- | --- |
| **Type of financial support** |  |  |  |  |
| Unemployment benefit | 11.8 (6) | 0.0 (0) | 5.3 (1) | 28.6 (2) |
| Income support | 19.6 (10) | 15.8 (3) | 10.5 (2) | 0.0 (0) |
| Integration assistance and refunds of travel expenses for disabled people and for special social needs | 60.9 (31) | 31.6 (6) | 68.5 (13) | 0.0 (0) |
| Disability pension | 5.9 (3) | 26.3 (5) | 73.7 (14) | 28.6 (2) |
| Care and housing allowence | 53 (27) | 52.6 (10) | 36.9 (7) | 42.9 (3) |
| Pension and Basic income for retired people | 41.2 (21) | 42.2 (8) | 31.6 (6) | 28.6 (2) |
| Child benefit | 33.3 (17) | 57.9 (11) | 89.5 (17) | 28.6 (2) |
| Others | 11.8 (6) | 10.5 (2) | 105.3 (20) | 14.3 (1) |
